# New Insight into Sorption Cycling Stability of Three Al-Based MOF Materials in Water Vapour

**DOI:** 10.3390/nano12122092

**Published:** 2022-06-17

**Authors:** Tadeja Birsa Čelič, Aljaž Škrjanc, Juan Manuel Coronado, Tomaž Čendak, Victor Antonio de la Peña O’Shea, David Pedro Serrano, Nataša Zabukovec Logar

**Affiliations:** 1 National Institute of Chemistry, Hajdrihova 19, 1000 Ljubljana, Slovenia; tadeja.birsa@gmail.com (T.B.Č.); aljaz.skrjanc@ki.si (A.Š.); tomaz.cendak@gmail.com (T.Č.); 2 School of Science, University of Nova Gorica, Vipavska 13, 5000 Nova Gorica, Slovenia; 3 IMDEA Energy Institute, Avenida Ramón de la Sagra, 3, Parque Tecnológico de Móstoles, Móstoles, 28935 Madrid, Spain; jm.coronado@csic.es (J.M.C.); victor.delapenya@imdea.org (V.A.d.l.P.O.); david.serrano@imdea.org (D.P.S.); 4 Chemical and Environmental Engineering Group, Rey Juan Carlos University, C. Tulipán, s/n, Móstoles, 28933 Madrid, Spain

**Keywords:** MIL-96(Al), MIL-100(Al), and MIL-110(Al), MOF, water sorption, cycling, hydrothermal stability

## Abstract

Three porous aluminium benzene-1,3,5-tricarboxylates MIL-96(Al), MIL-100(Al) and MIL-110(Al) materials were studied for their hydrothermal stability. The 40-cycles water vapour sorption experiments for the three samples were performed by varying the temperature between 40 and 140 °C at 75% relative humidity to simulate working conditions for materials used in water sorption-based low-T heat storage and reallocation applications. The materials were characterized by powder X-ray diffraction, N_2_ physisorption, and Nuclear Magnetic Resonance and Infrared spectroscopies before and after the cycling tests. The results showed that the structure of MIL-110(Al) lost its crystallinity and porosity under the tested conditions, while MIL-96(Al) and MIL-100(Al) exhibited excellent hydrothermal stability. The selection of structures, which comprise the same type of metal and ligand, enabled us to attribute the differences in stability primarily to the known variances in secondary building units and the shielding of potential water coordination sites due to the differences in pore accessibility for water molecules. Additionally, our results revealed that water adsorption and desorption at tested conditions (T, RH) is very slow for all three materials, being most pronounced for the MIL-100(Al) structure.

## 1. Introduction

Metal organic frameworks (MOFs) with structures built from inorganic building units connected to multi-dentate organic ligands belong to a group of crystalline nanoporous materials [1]. Due to their unique structural properties (high surface areas, low framework densities, well defined pore dimensions and shapes) and ability to design their structural and chemical properties, MOFs have shown a great potential in different processes such as adsorption, catalysis, medical applications, magnetism, luminescence, gas separation, etc. [2,3,4,5]. In recent years special attention was devoted to the applicability of MOFs as adsorbents in areas related to sustainable development, i.e., separation/selective adsorption of CO_2_ over other gases [6] and in low-temperature heat transformation applications (thermally driven heat pump and/or chillers) based on the reversible adsorption/desorption of water [7,8], to name only a few. The latter stimulated an intensive research on their water sorption properties and function, as well as on their sensitivity or instability in the presence of moisture and other agents [9,10,11]. To date, numerous studies on water sorption in MOFs from the heat-reallocation-applications viewpoint of view have been published [12,13,14,15,16,17], with a recent emphasis on aluminium MOFs as environmentally benign and stable MOFs [15,16,17,18].

A detailed study of the influence of structural features on the hydrothermal stability of porous coordination polymers was first published by Low et al. [19]. The predictions of this work were further supported by investigations of structural stability of the other MOF systems (e.g., MOF-5, MOF-177, IRMOF-1, HKUST-1, UMCM-150, MIL-100, ZIF-8, ZIF-11, DMOFs) [20,21,22,23,24]. From these studies, it can be concluded that the stability of MOFs in the presence of moisture is related to a variety of factors. These include the strength of the metal-ligand bonds or the basicity (*p*K_a_) of the ligand, the oxidation and coordination state of the metal, the dimensionality of the framework and secondary building units (SBU), the type of connection node between the metal centres and the electron donor group, the catenation of the framework, and also weaker intermolecular interactions (H-bonds, π-π interactions, etc.) [10,21,24,25,26,27]. To date, there have also been numerous attempts to develop new strategies to increase the water resistance of existing MOFs. The incorporation of hydrophobic functional groups on the organic ligands has proven to be one of the most efficient methods [11,19,28,29]. Moreover, the preparation of composites with hydrophobic polymers proved to be another approach to increase the hydrothermal stability of MOFs (e.g., MIL-100(Fe), HKUST-1) [28,30,31].

We have focused on the investigation of the water adsorption cycling stability of three Al MOFs: MIL-96(Al)[Al_12_O(OH)_18_(H_2_O)_3_(Al_2_(OH)_4_)(BTC)_6_·nH_2_O] [32], MIL-100(Al) [Al_3_O(OH)(H_2_O)_2_(BTC)_2_·nH_2_O] [33] and MIL-110(Al) [Al_8_(OH)_12_{(OH)_3_(H_2_O)_3_}(BTC)_3_·nH_2_O] [34] (BTC = benzene-1,3,5-tricarboxylic acid) at conditions suitable for low temperature (solar) heat storage and reallocation. Furthermore, the aim of the research was to evaluate the role of variations in the SBUs, including the degree of metal-ligand connectivity and steric shielding of possible water coordination sites in the three MOFs, on their structural stability under selected humid conditions. While the tested MOFs have already been widely investigated for a variety of properties and applications, e.g., CO_2_ capture, separation, energy storage, cooling, precursors for mesoporous Al_2_O_3_ formation, etc. [35,36,37,38,39,40,41,42,43], the role of the geometry of SBUs and associated topology on their hydrothermal stability was not evaluated so far for the selected three structures.

## 2. Materials and Methods

### 2.1. Materials

Aluminium nitrate nonahydrate (Al(NO_3_)_3_·9H_2_O, Merck, Darmstadt, Germany, 98.5%), benzene-1,3,5-tricarboxylic acid (C_9_H_6_O_6_, Aldrich, St. Louis, MO, USA, 95%) (BTC), trimetilbenzene-1,3,5-tricarboxylate ((CH_3_CO_2_)_3_C_6_H_3_, Aldrich, St. Louis, MO, USA, 98%), nitric acid (HNO_3_, Merck, Darmstadt, Germany, 65%), *N*,*N*-dimethylformamide (C_3_H_7_NO, Aldrich, St. Louis, MO, USA) were obtained commercially and used without further purification. Water was deionized in-house.

MIL-96(Al) was synthesized according to the procedure in the literature [32]: the reaction mixture of Al(NO_3_)_3_·9H_2_O (3.5 mmol), C_9_H_6_O_6_ (0.5 mmol) and H_2_O (278 mmol) was placed in a Teflon-lined autoclave, which was sealed and heated at 210 °C in an oven for 24 h. The resulting white powder was collected by filtration, washed with deionized water, and dried at room temperature.

Synthesis and activation of MIL-100(Al) were performed as suggested by Volkringer et al. [33]. The reaction mixture of Al(NO_3_)_3_·9H_2_O (1.5 mmol), (CH_3_CO_2_)_3_C_6_H_3_ (1 mmol), HNO_3_ (1.9 mmol) and H_2_O (380 mmol) was placed in a Teflon-lined autoclave and heated at 210 °C for 3.5 h. The as-synthesized yellowish compound was filtered, washed with deionised water, and dried. The samples were activated with soaking in DMF in Teflon autoclaves (150 °C, 4 h) and with water under reflux conditions (100 °C, 12 h). The white MIL-100(Al) was vacuum filtered and dried in air at room temperature.

MIL-110(Al) was also synthesized hydrothermally according to the literature [34]. The highly acidic reaction mixture of Al(NO_3_)_3_·9H_2_O (1.8 mmol), (CH_3_CO_2_)_3_C_6_H_3_ (1.2 mmol), HNO_3_ (4 mmol) and H_2_O (278 mmol) was placed in a Teflon-lined autoclave and after crystallization in an oven at 210 °C (72 h), the resulting powder was filtered and dried at room temperature. The as-synthesized material was activated by stirring in deionized water under reflux at 100 °C for 16 h, vacuum filtered and left to dry in air and room temperature.

### 2.2. Methods

Powder X-ray diffraction (PXRD) was performed on a PANalytical X’Pert PRO diffractometer (Malvern Panalytical, Almelo, The Netherlands), with Cu Kα radiation (λ = 1.5406 Å). The XRD patterns were collected using continuous scanning mode in 2*θ* range 5–60° with a scanning step of 0.016° at a counting time of 100 s per step.

The combined TG/DTA measurements were carried out in an argon flow (10 mL∙min^−1^) with a heating rate of 10 °C∙min^−1^ from 25 °C to 700 °C using a thermal analysis system TGA Q5000IR (TA Instruments, Inc., New Castle, DE, USA).

Microstructural analysis was performed by Zeiss SUPRA^TM^ 35 VP scanning electron microscope (SEM) with a field emission gun (Carl Zeiss Microscopy, Jena, Germany).

The textural properties of materials were characterized from N_2_ isotherms measured at 77 K on a ASAP 2020 volumetric adsorption analyzer (Micromeritics, Norcross, GA, USA). Before measurements, the sample MIL-100(Al) was activated by degassing at 200 °C under high vacuum for 12 h, while samples MIL-96(Al) and MIL-110(Al) were degassed for 12 h at 150 °C. These temperatures were selected based on the TGA analysis and literature review. The Bruauner–Emmett–Teller surface areas were estimated using the adsorption data in a relative pressure ranging from 0.05 to 0.12 according to the BET equation. The micropore volumes were calculated using Dubinin–Astakhov equation and the total pore volumes were determined as the volume of liquid nitrogen adsorbed at a relative pressure of p/p_0_ = 0.95.

^13^C and ^27^Al NMR spectra were recorded on the 600 MHz Varian system using 1.6 mm NB Triple Resonance HXY FastMAS probe (Agilent Technologies, Santa Clara, CA, USA). Chemical shifts were referenced relative to signals in tetramethylsilane and 1 M aqueous Al(NO_3_)_3_ for ^13^C and ^27^Al, respectively. ^1^H-^13^C CPMAS (cross-polarization magic angle spinning) spectra were recorded with 3 s of relaxation delay and 5 ms of contact time for RAMP [44] cross-polarization. ^27^Al single pulse spectra were measured using 17 μs long π/2 pulses and 250 ms of relaxation delay. Samples were spun with the frequency of 20 kHz and XiX [45] proton decoupling was used for both, ^13^C and ^27^Al, measurements.

DRIFTS (Diffuse Reflectance Infrared Fourier Transform Spectroscopy) experiments were carried out in a Thermo Nicolet 5700 FTIR spectrometer (Thermo Fisher Scientific, Waltham, MA, USA) equipped with a MCT detector cooled with liquid N_2_, and a Harrick high-temperature cell provided with BaF_2_ windows. A continuous gas flow rate of 50 mL∙min^−1^ of humid air (ca. 80% RH checked with an online Vaisala humidity sensor) was used in all these assays. The spectra were recorded at temperatures between 50 and 200 °C by accumulation of 64 scans at a resolution of 4 cm^−1^.

The water vapour adsorption/desorption isotherms and water cycle measurements were performed on an IGA-100 gravimetric analyzer (Hiden Isochema Ltd., Warrington, UK). Prior to H_2_O isotherms, the samples were in situ outgassed at the same conditions as above detailed for the nitrogen physisorption analyses in order to remove solvent molecules. Adsorption isotherms for water vapour were measured at 25 and 40 °C with an equilibrium time of 80 min for all measurements. A typical water sorption 40-cycles experiment was performed at 56 mbars with a flow rate of 10 mL∙min^−1^, the materials being firstly dried at 150 and 200 °C in dry helium flow (0% RH). The relative humidity, which was adjusted by using two mass flow controllers that varies the ratio of saturated and dry helium, was then set to 75% and the sample temperatures were varied between 40 and 140 °C. The water loading capacity of materials was measured at the beginning, after 20 and after 40 cycles. For these three listed cycles, the duration of the measurements was 24 h and for the rest of the cycles 5 h.

The simulation of the Connolly surface and total accessible solvent surface was calculated using Materials Studio simulation package 4.0 (Accerlys software, San Diego, CA, USA). Water was selected as probe molecule, with a Van der Walls radius of 1.4 Ǻ. The calculations were carried out in an Intel tetra-processor Xeon Work Station with 24 GB RAM.

## 3. Results and Discussion

### 3.1. Water Sorption Capacity

**Figure 1:**
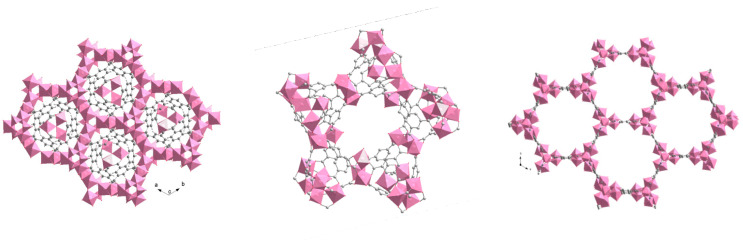
Schematic representation of the MIL-96(Al), MIL-100(Al) and MIL-110(Al) crystal structures (from left to the right). AlO_6_ octahedra (magenta), C atoms (grey) and O atoms (red). Structures are drawn from the CIF-files by using DIAMOND program [46]. MIL-96(Al) and MIL-100(Al) both comprise tri-nuclear Al-oxo clusters and MIL-96(Al) additional sinusoidal chains of aluminium octahedra. MIL-110(Al) exhibits unique octanuclear Al-oxo clusters.

The pure phases of the materials MIL-96(Al), MIL-100(Al) and MIL-110(A) (Figure 1) were prepared based on literature data and investigated for their water sorption capacity.

The capacity was initially estimated by thermogravimetry (TG) since it is considered as one of the simplest and the most popular methods for water content determination in sorbents [47]. Before TG analysis, the samples were placed in a desiccator with 75% relative humidity at 25 °C for 7 days.

**Figure 2:**
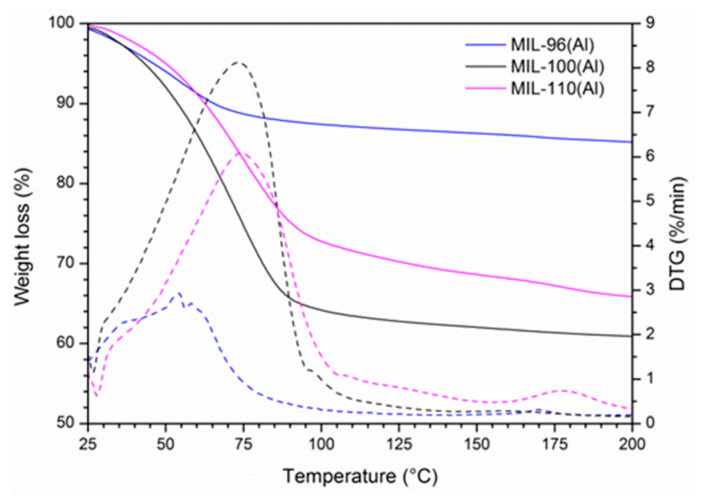
TG (solid) and DTG (dash) curves of MIL-96(Al), MIL-100(Al) and MIL-110(Al) materials.

The TG curves (Figure 2) for all three compounds showed large weight losses: 13.7 wt.% for MIL-96(Al) up to 150 °C, 31.4 wt.% for MIL-110(Al) up to 150 °C and 39.1 wt.% for MIL-100(Al) up to 200 °C. We assume that this loss is due to the removal of adsorbed water molecules. The presence of other occluded moieties, like traces of BTC ligand, in activated MIL-110(Al) has also been reported [48]; however, their degradation is reported to occur above 300 °C. By dividing the weight losses due to water desorption by the weight of the dry samples, the water capacity of investigated materials was determined. The MIL-100(Al) showed the largest water capacity (0.64 g∙g^−1^), followed by MIL-110(Al) (0.46 g∙g^−1^) and MIL-96(Al) material (0.16 g∙g^−1^).

**Figure 3:**
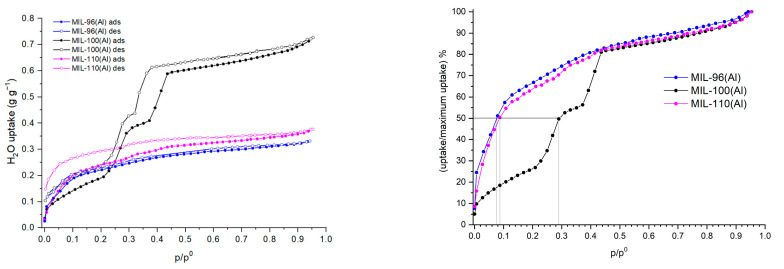
Water sorption isotherms of MIL-96(Al) (blue), MIL-100(Al) (black) and MIL-110(Al) (magenta) at 313 K (**left**) and ratio of water uptake to its maximum value in function of relative pressure (**right**). p/p^0^ is the relative pressure of water with p^0^ = 7.383 kPa (73.833mbar). Solid and open symbols denote adsorption and desorption, respectively.

Figure 3 (left) shows water adsorption and desorption isotherms for MIL-96(Al), MIL-100(Al) and MIL-110(Al). The water sorption properties of MIL-100(Al) have already been studied [34,49].In our study, MIL-100(Al) adsorbs more water (0.73 g of H_2_O per g of dry sorbent at a relative pressure of 0.95) than previously reported [42]. However, the mechanism of pore filling and a small hysteresis between adsorption and desorption branches, due to the capillary condensation, framework flexibility and hydrophilicity of the framework [50], are comparable. The maximum water uptake of MIL-96(Al) is 0.33 g∙g^−1^ and for MIL-110(Al) 0.38 g∙g^−1^ at relative pressure 0.95. In the case of MIL-96(Al), there is also no evident hysteresis, while the desorption branch of MIL-110(Al) is shifted upward in the lower pressure region. As can be seen in Figure 3 on the left, the shapes of MIL-96(Al) and MIL-110(Al) water isotherms are similar and both materials exhibit type-I isotherms. On the other hand, MIL-100(Al) exhibits a type-IV like isotherm, where it adsorbs the largest amount of water in the relative pressure range between 0.1 and 0.4. The isotherms show that MIL-96(Al) and MIL-110(Al) are more hydrophilic than MIL-100(Al) as they adsorb 50% of the maximum uptake below relative pressure of 0.1 (Figure 3 right). The water adsorption isotherm of MIL-100(Al) indicates that the water molecules first attach to the very hydrophilic sites, most likely to the hydroxyl groups on the framework and then the consecutive filling of the pores takes place. The differences in the water content determined from the water sorption isotherms and estimates from TG measurements are due to the different hydration regimes (before/during measurements) for the two methods.

**Figure 4:**
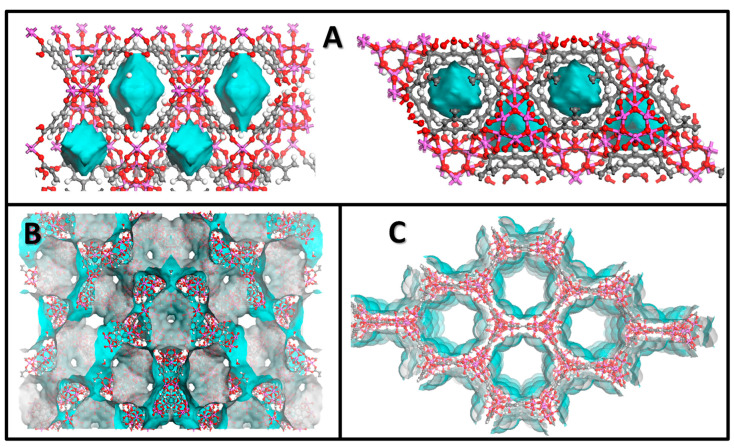
Representation of accessible water surface (blue) for (**A**) MIL-96(Al), (**B**) MIL-100(Al) and (**C**) MIL-110(Al) adsorbents.

Additionally, the Connolly and solvent accessible surfaces (Figure 4) were calculated for each sample. While the pores of MIL-96 (Al) are almost completely covered by a water monolayer (with only 0.07 cm^3^∙g^−1^ of free volume), both MIL-100 (Al) and MIL-110 (Al) have more than 40% of free volume. Considering these data, we calculated that the theoretical uptake of a water monolayer for MIL-96(Al), MIL-100(Al) and MIL-110(Al) is 0.19 g∙g^−1^, 0.35 g∙g^−1^ and 0.34 g∙g^−1^, respectively. We also determined the maximum theoretical water uptake of 0.26 g∙g^−1^ for MIL-96(Al), 1.01 g∙g^−1^ for MIL-100(Al) and 0.95 g∙g^−1^ for MIL-110(Al). The calculated data for MIL-96(Al) are mostly in agreement with the data obtained from the water isotherms since the theoretical values are only slightly lower than the experimental ones. In the case of MIL-100(Al), the simulation confirms the highest water adsorption capacity among the studied materials. The difference between the calculated and the measured maximum water uptake could be the result of formation of ordered clusters in the vicinity of the hydrophilic metal sites, which consequently prevent the complete filling of the entire free volume in the pores with water molecules. A second possible explanation is that the activation of the materials is not complete at the selected conditions. On the other hand, a significant deviation between experimental and simulated data was observed in the case of the MIL-110(Al) material. The measured maximum water uptake (0.38 g∙g^−1^) corresponds to the theoretical uptake of a water monolayer, which could suggest that in a real system the channels of MIL-110(Al) are filled mainly with a monolayer of water molecules. It could be assumed that, when the monolayer of water molecules is formed, the adsorption of additional water molecules does not occur. A detailed insight into the desorption branch of the water isotherms of MIL-110(Al) showed that in the lower relative pressure region the curve is shifted upward indicating slower diffusion of water molecules from channels, most probably due to the presence of strong hydrogen bonds formed between framework water/OH groups and sorbed water molecules. Alternative explanations for the lower experimental water uptake include the possible presence of extra-framework BTC that hinders the water uptake and/or repulsive forces between water molecules and exposed hydrophobic benzene functionalities that are located along the large channels in MIL-110(Al). The last possible explanation could be partial degradation of the framework already during the water sorption experiment. 

Complete water sorption characterization of porous material includes, in addition to the measurements of water isotherms, a calculation of the heat of adsorption which can be determined either from two adsorption isotherms measured at different temperatures by applying a modified form of the Clausius–Clapeyron equation or from one adsorption isotherm by using the Dubinin–Raduschevich (DR) equation. In this study, the DR analysis [51] was used to calculate the isosteric heat of adsorption and to analyse the pore filling with vapours. 

Three different values of *q_st_* for each isotherm were estimated from the H_2_O sorption data measured at 313 K (see Table S1). From calculated *q_st_* values it is evident that, in all three materials at low relative pressure region (p/p_0_ < 0.2), strong interactions between the adsorbed water molecules and the framework are present, which can be assigned to adsorption of water on hydrophilic sites (OH or framework water). With increasing relative pressure *q_st_* values gradually drop. However, isosteric heat of adsorption for the MIL-96(Al) and MIL-110(Al) materials at higher relative pressures is larger in comparison with MIL-100(Al). This is in agreement with the fact that in microporous materials with small pores, interactions between the majority of water molecules and/or framework are stronger than interactions between H_2_O molecules in disordered clusters formed in larger pores at higher relative pressures.

### 3.2. Cycling Test

**Figure 5:**
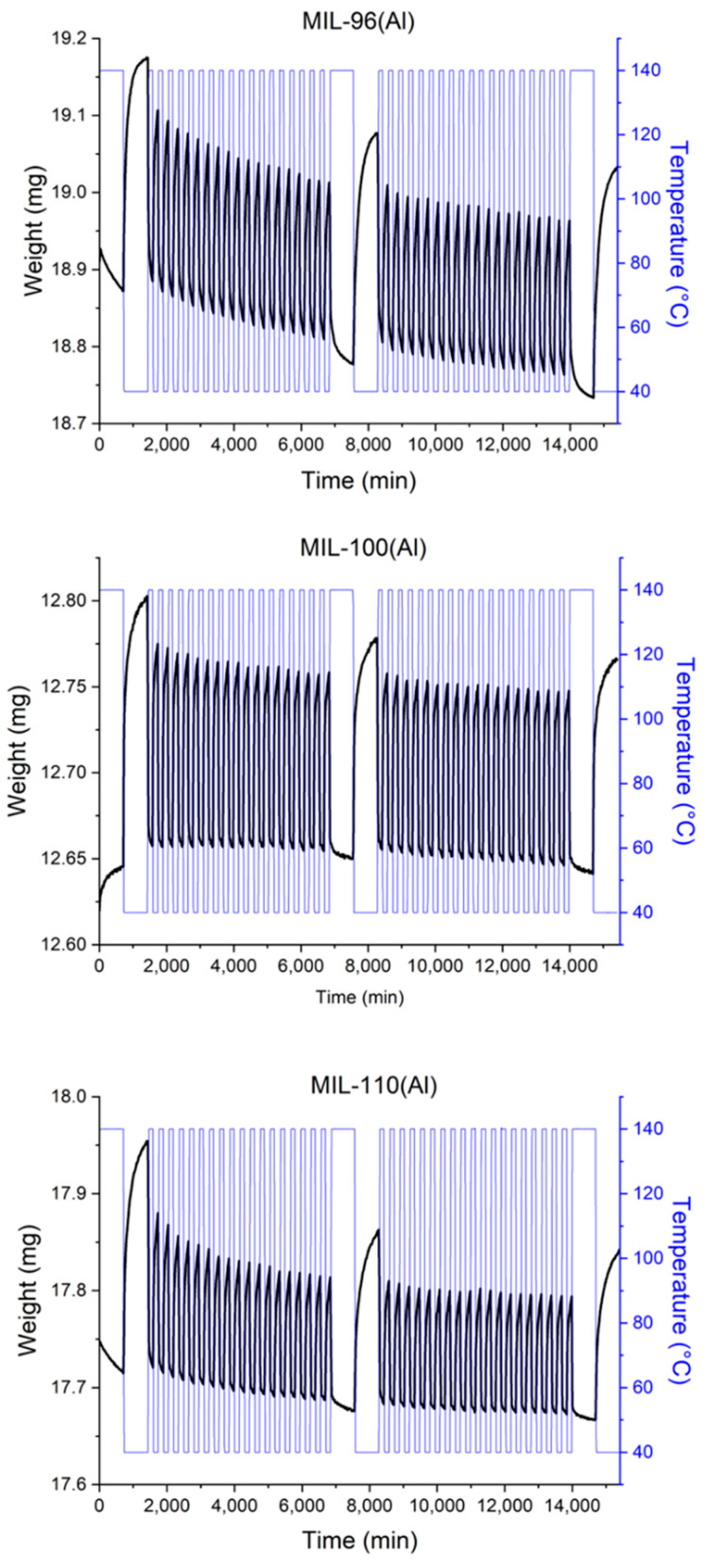
Hydrothermal cycling treatment of water adsorption/desorption for MIL-96(Al), MIL-100(Al) and MIL-110(Al) materials between temperatures of 40 °C and 140 °C at 75% relative humidity under a helium gas flow.

The cycling tests with 40 consecutive adsorption/desorption cycles was performed by treating the samples in a water vapour atmosphere at 56 mbar and 75% RH and by varying the temperature from 40 to 140 °C. The results of continuous cycling stability tests on the three studied MOFs are shown in Figure 5 and Table 1.

As can be seen from the results at the beginning, in the middle and at the end of the cycling treatment, the water loading capacity of MIL-110(Al) decreased by 19% over 20 cycles and by 25% over 40 cycles. For MIL-100(Al) material, the numbers are slightly different, i.e., the water loading capacity decrease by 19% over 20 cycles and by 20% over 40 cycles. The cycling stability of MIL-100(Al) has been already examined by Jeremias and co-workers, where a smaller, but gradual, loss of water loading was reported after 20 and 40 cycles (4.5% and 6.6%, respectively). However, the maximum water uptake was lower than in our case (more than 30%), which was explained by the possible presence of unremoved ligand in the pores, inhibiting complete wetting of the pore walls [42]. Here, the water capacity seems to stabilize after 20 cycles. In the case of MIL-96(Al) material, the maximum water loading decreased only by ca. 1% over 40 cycles. Similar cycling tests on the MIL-96(Al) and MIL-100(Al) composites on Al support reported by Yang et al. [38] showed a decrease of 10% over 20 cycles.

### 3.3. Structural Characterization of Materials

#### 3.3.1. PXRD and SEM Analysis

To understand water sorption performance and cycling stability, each MOF was characterized by using PXRD and SEM before and after cycling tests. The PXRD data (Figures S1 and S2) showed that MIL-96(Al), MIL-100(Al) and MIL-110(Al) crystalline phases were successfully synthesized. Furthermore, PXRD indicated that MIL-96(Al) and MIL-100(Al) retained their crystallinity after 40-cycling treatment. In the MIL-96(Al) sample after the cycling test, traces of AlO(OH) could be detected. On the other hand, MIL-110(Al) completely transformed to AlO(OH) phase. The morphology of the as-synthesized materials and that of the samples after the 40-cycling treatment was checked by SEM (Figure S3). The MIL-96(Al) morphology is characterized by hexagonal crystals (3 µm), while the SEM images of MIL-100(Al) and MIL-110(Al) materials show octahedral shaped crystals and hexagonal rod-shaped crystals, respectively. SEM image of MIL-110(Al) after 40-cycling treatment revealed that despite the PXRD showing amorphization of the material, the hexagonal rod-shaped morphology was maintained, with the formation of a second phase evident in the SEM images, identified as AlO(OH).

#### 3.3.2. N_2_ Physisorption

N_2_ adsorption isotherms at 77 K were measured to evaluate the textural properties of the materials (Figures S4 and S5). The isotherms of all three MOFs show type-I curves, which are characteristic for microporous materials and are consistent with previous reports for the three materials. The micropore volumes estimated by applying the Dubinin–Astakhov equation are 0.12 cm^3^∙g^−1^ for MIL-96(Al), 0.57 cm^3^∙g^−1^ for MIL-100(Al) and 0.32 cm^3^∙g^−1^ for MIL-110(Al). Considering micropore and total pore volumes (Table 2), as well as a pore size distribution graph (Figure S4) it is clear that all studied aluminium MOFs are microporous, with a small amount of mesopores in MIL-100(Al). 

The textural properties of the fresh materials resemble some of the previously reported values [33,35,52]. It must be emphasized here that N_2_ physisorption has proven questionable for determination of textural properties of the narrow-pore MIL-96(Al), because the pore entrance diameter of the material is too small for nitrogen molecules to freely enter the pores, so the data must be viewed with caution [53]. The increase in BET surface area of MIL-100(Al) after water sorption is most probably due to incomplete activation of the sample prior to the cycling experiment. The loss of porosity of MIL-110(Al) after cycling is consistent with the loss of crystallinity.

#### 3.3.3. Solid State NMR

**Figure 6:**
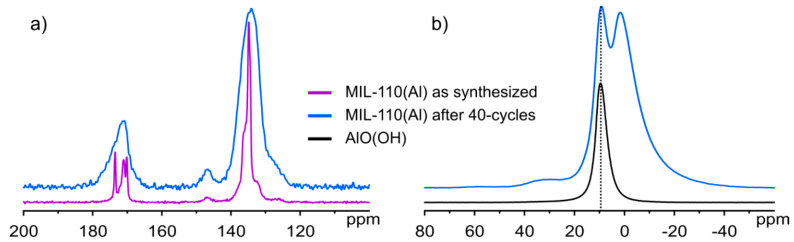
(**a**) ^1^H-^13^C CPMAS NMR spectra of MIL-110(Al) before and after cycling test; (**b**) ^27^Al single pulse NMR spectra of AlO(OH) and MIL-110(Al) after 40-cycles.

^1^H-^13^C CPMAS spectra of MIL-110(Al) before and after the cycling test (Figure 6a) show that the framework structure changed as hinted by significantly different carbon spectra. Namely, the as-synthesized sample exhibits narrow carbon signals in the aromatic and carboxyl regions while the same signals are strongly broadened after the cycling test, thus suggesting that the crystallinity of the sample was lost. To further inspect what happens during the cycling test, ^27^Al single pulse NMR measurements were performed. Firstly, the spectrum of AlO(OH) was recorded to serve as a reference and, as depicted in Figure 6b, it exhibits a single aluminium peak at 9.6 ppm. A peak resonating at the same frequency can also be observed in the spectrum of MIL-110(Al) after 40-cycles, suggesting that the cycling test indeed causes the formation of AlO(OH), which is in agreement with PXRD results.

**Figure 7:**
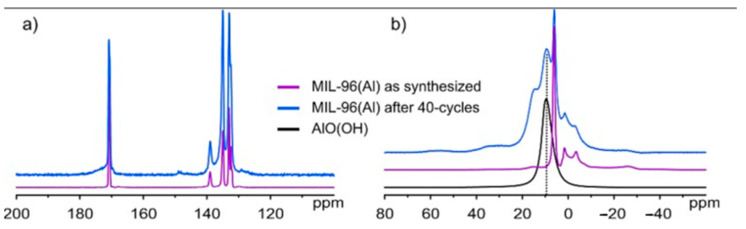
(**a**) ^1^H-^13^C CPMAS NMR spectra of MIL-96(Al) before and after cycling test; (**b**) ^27^Al single pulse NMR spectra of AlO(OH), as-synthesized MIL-96(Al) and MIL-96(Al) after 40-cycles.

In order to obtain additional insights, ^13^C and ^27^Al NMR measurements were performed on MIL-96(Al) samples (Figure 7). ^1^H-^13^C CPMAS spectra of MIL-96(Al) before and after the cycling test (Figure 7a) confirmed the XRPD results; MIL-96(Al) remains structurally intact as proven by almost identical carbon spectra with only slight broadening of peaks observed in the spectrum of cycle-tested sample. Additionally, aluminium single pulse NMR measurements (Figure 7b) revealed that the distinct signal, corresponding to AlO(OH) and resonating at 9.6 ppm, could also be detected in cycle-tested MIL-96(Al) whereas such a signal is not observed in the spectrum of the as-synthesized sample. However, NMR measurements could not provide any information on where the AlO(OH) phase is located.

With regard to the obtained NMR results and detailed comparison of the XRPD patterns of the as-synthesized MIL-96(Al) material before and after the cycling test (Figure S1), which also revealed the presence of a small amount of the boehmite AlO(OH) phase after cycling, we concluded that the decrease of the MIL-96(Al) porosity characteristics is not the result of the complete sample degradation. The inaccessibility of the pores is more likely the result of the formation of a thin layer of additional AlO(OH) phase which hinders the access to the interior of the already narrow pores.

The alignment of structural properties and the water sorption performance for MIL-100(Al) show that the increase of water uptake after the cycling test should be assigned to a possible rearrangement/removal of small amount of extra-framework species, blocking the pores, since the crystallinity after the test was comparable to the crystallinity of as-synthesized material and since its BET surface area and micropore volume increased after cycling (Table 2). The mobility of extra-framework species (water and traces of BTC) was already reported by Haouas et al. [54] Additionally, our results indicate that the kinetics of water adsorption and desorption in cycling treatment of all three investigated materials is very slow and the equilibrium in individual cycles was not reached even after 12 h (Figure 5). The slow diffusion of water molecules from MIL-100(Al) leads to it having the smallest water loading capacity in dynamic conditions in contrast to it having the largest in isothermal measurement conditions. The discrepancy can be further explained by the fact that heating up to 140 °C does not lead to desorption of more tightly bonded water, e.g., the molecules coordinated to Al trimers. Furthermore, in the MIL-100 structure, the large cavity is surrounded by smaller pores, which additionally hinder and slow down desorption [36].

MIL-110(Al) was considered in the literature as very stable in humid conditions [19]. However, we found that in our experimental conditions its structure collapsed leading to decrease of water sorption performance. Namely, XRPD patterns of MIL-110(Al) material (Figure S1), show that the framework did not retain its structure after 40 cycles since the formation of AlO(OH) was observed. XRPD results were also confirmed by solid-state NMR measurements, as discussed above. 

In the MIL-96(Al) sample, water vapour exposure did not lead to the collapse of the structure, although some loss of crystallinity can be deduced from the XRPD patterns, as mentioned above (Figure S1). Nevertheless, the BET surface area of MIL-96(Al) after 40 cycles decreased from 310 m^2^∙g^−1^ to 50 m^2^∙g^−1^ (ca. 80% reduction) and the micropore volume was reduced from 0.12 to 0.02 cm^3^∙g^−1^ (Table 1). As confirmed by NMR analysis, the formation of small amount of boehmite AlO(OH) phase on the surface, also detected by XRPD, after the cycling experiment is most probably responsible for the reduced BET; however, it does not influence the water sorption performance.

### 3.4. FTIR Study

To further evaluate hydrothermal stability of MIL-96(Al), MIL-100(Al) and MIL-110(Al), DRIFT spectroscopy was utilized to monitor the interaction of water molecules with aluminium trimesate frameworks under dynamic conditions. Accordingly, the adsorption-desorption process of water was investigated by means of DRIFT in a stream of humid air (ca. 80% RH) in four cooling and heating cycles in the temperature range from 50 °C to 200 °C. These experiments are similar to those performed in the thermogravimetric study of the dehydration-rehydration cycles and they can provide complementary insights into the nature of the preferential centres for water adsorption.

**Figure 8:**
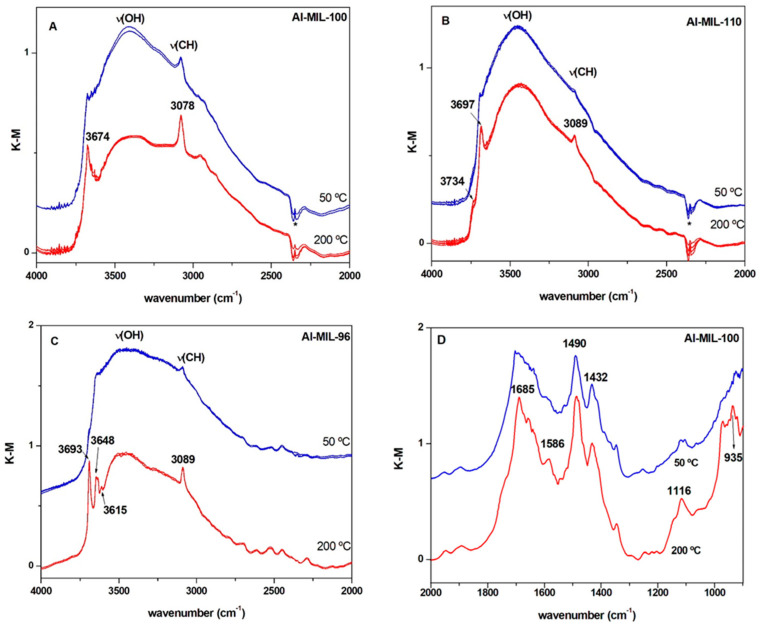
DRIFT spectra of following successive cycles of heating at 200 °C (red) and cooling at 50 °C (blue) in a stream of humid air of (**A**) MIL-100(Al), (**B**) MIL-110(Al) and (**C**) MIL-96(Al) materials. The overlapping of the spectra corresponding to four cycles indicates the good reproducibility of this thermal treatment. Panel (**D**) shows the DRIFT at lower wavenumber range, showing the structural bands of the linker, for MIL-100(Al) at 200 °C and 50 °C in wet airflow. The asterisk (*) marks the band of atmospheric CO_2_ incompletely removed.

The characteristic bands of the linker vibrations appear below 2000 cm^−1^, but the present study focuses on the features associated to the OH stretching of the water molecules, which are observed in the 3700 and 2500 cm^−1^ range [55,56]. In addition, spectra in the near infrared region can also be useful to follow water adsorption by monitoring (ν + δ) combination bands of water and hydroxyl groups, and this is illustrated in Figure S6 for MIL-96(Al) [53]. Figure 8 shows the DRIFT spectra in this region of the three samples of aluminium trimesate following several cycles of heating to 200 °C and cooling to 50 °C in a flow of humid air. This treatment allows switching between water loaded and water depleted states of the samples, which can be easily distinguished by DRIFTS. The spectra of the three materials show a nearly perfect reproducibility following a few of these cycles of sorption-desorption, highlighting the hydrothermal stability of the materials. In the case of MIL-100(Al) (see Figure 8A), the very broad envelope centred at about 3415 cm^−1^ for the treatment at 50 °C is related to hydrogen-bonded water molecules present during the pore filling step [57]. Heating the sample at 200 °C for 10 min results in a significant decrease of the intensity of this broad feature, and simultaneously a sharper band grows at 3674 cm^−1^. This last feature can be associated to terminal OH coordinated to the aluminium clusters, which becomes evident due to the desorption of H-bonded water molecules, as reported previously for MIL-96(Al) [53]. The relatively sharp band at 3078 cm^−1^, which appears after the high temperature treatment, corresponds to the CH stretching of the aromatic rings of the linker. Apart from the changes in intensity, this feature is not significantly affected by thermal treatment, suggesting that the interactions of water molecules do not significantly modify the linker. Likewise, structural bands related to the linker, and present below 2000 cm^−1^ (see Figure 8D), are mostly unaffected by water adsorption for the three MOFs, although some sharpening of bands associated with C=O stretching (several bands at around 1650 cm^−1^ and 1490 cm^−1^) are observed after heating. Similarly, the increase of the intensity of bands of C–H in-plane bending modes (1116 cm^−1^) and those from OH deformation mode (around 950 cm^−1^), together with those already mentioned of the C-H stretching bands (3078 cm^−1^), are due to the removal of water molecules weakly interacting with the organic framework. On the other hand, although the treatment at 200 °C in humid air cannot remove completely sorbed water for any of the considered materials, judging by the decrease in the intensity of the broad band at around 3430 cm^−1^, MIL-100(Al) appears to interchange a relative larger amount of water, in agreement with the adsorption isotherms.

The spectra of the MIL-110(Al) (Figure 8B) show a very similar evolution with heating. Thus, at 200 °C the intensity of the broad band of adsorbed water molecules decreases and simultaneously the band at 3687 cm^−1^ increases, revealing terminal OH groups coordinated to aluminium centres. Besides, a shoulder at 3734 cm^−1^ can also be seen after thermal treatment, and it could be related to Al-OH species, akin to those found in alumina [53]. Finally, after heating, a clear C-H stretching band develops at 3089 cm^−1^. 

Figure 8C shows the DRIFT spectrum of MIL-96(Al) which is similar to those of the other samples at 50 °C (Figure 8A,B), but develops additional features after the treatment at 200 °C. In the spectrum of MIL-96(Al) heated to 200 °C, a narrow and relatively isolated band at 3693 cm^−1^ and two other sharp bands at 3642 and 3615 cm^−1^ appear. According to previous reports, the peak at the higher wavenumber can be associated with terminal Al-OH centres, whereas the other two features at lower wavenumber can be assigned with different bridging hydroxyls [53]. On the other hand, the broader band at 3521 cm^−1^ is assigned to structural water molecules. Considering the structure of MIL-96(Al), this spectral feature could be associated with the water molecules connected to the oxo-bridges by hydrogen bonding.

These results show that, despite structural differences, for these three MOFs, cycles of adsorption-desorption induced by heating involve mainly the interchange of water molecules attached by hydrogen-bonding to OH groups coordinated to Al centres, with relatively little interaction with the organic framework.

### 3.5. Structure-Property Relationship

Since the hydrothermal stability of metal-organic framework materials depends on several structural parameters, Jasuja [21] and co-workers first proposed to keep as many of these variables as possible constant in order to better specify which of them has a decisive impact. By selecting three aluminium trimesates we have focused on determining the correlations between their hydrothermal stability and the nature of the secondary building units, as well as the pore size distribution, since in all three materials the oxidation state of aluminium is 3+, it is octahedrally coordinated and the trimesate linker (BTC) is bonded in a *η*^1^ fashion to aluminium cations.

In MIL-96(Al) [Al_12_O(OH)_18_(H_2_O)_3_(Al_2_(OH)_4_)(BTC)_6_·nH_2_O], the framework is built up from isolated trinuclear *µ*_3_-O aluminium complexes AlO_5_(H_2_O) and 18-member chains of corner sharing AlO_4_(OH)_2_ and AlO_2_(OH)_4_ octahedra forming cavities up to 9 Å in diameter. MIL-100(Al) with empirical formula [Al_3_O(OH)(H_2_O)_2_(BTC)_2_·nH_2_O] consists of *µ*_3_-oxocentered trinuclear units of aluminium octahedra connected via BTC ligand and comprise 5.2 Å and 8.8 Å channels opening to 25 Å and 29 Å cavities, respectively. In MIL-110(Al) [Al_8_(OH)_12_{(OH)_3_(H_2_O)_3_}(BTC)_3_·nH_2_O] the framework contains aluminium octanuclear clusters that are linked by BTC forming large 16 Å channels. The octanuclear motifs is composed of three dinuclear subunits of AlO_2_(OH)_3_(H_2_O) or AlO_2_(OH)_4_ edge-shared octahedra, linked by corners to two AlO_3_(OH)_3_ octahedra.

**Figure 9:**
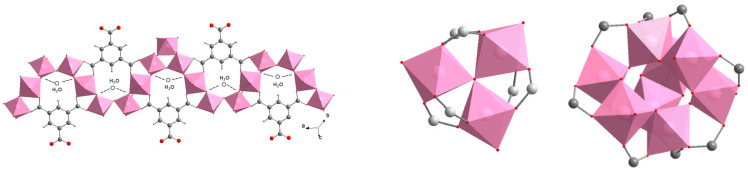
Presentation of the inorganic chain and hydrogen bonds in MIL-96(Al)^24^ (**left**), trimers in MIL-100(Al) (**middle**) and octanuclear clusters in MIL-110(Al) (**right**). AlO_6_ octahedra are rose, C atoms grey, O atoms red and H atoms light grey. Structures are drawn from the cif-files by using DIAMOND software [46].

Considering the obtained results and the structural characteristics of the studied materials (Figure 1 and Figure 9), we concluded that the stability of MIL-96(Al) under selected humid conditions is most probably due to the presence of corrugated chains of corner-sharing aluminium octahedra that are further stabilized by hydrogen bonds between water molecules from the pores and *µ*_2_-OH groups bridging the aluminium atoms. This type of steric shielding based on infinite SBUs has already been proven as one of key features in designing water stable MOFs [58]. Additionally, the discrete trinuclear units, which are connected to the chains of the aluminium octahedra through the BTC ligand, also contribute to the hydrothermal stability of MIL-96(Al) by further shielding the access of sorbed water molecules, i.e., preventing the attack on the metal-ligand bond.

On the other hand, the frameworks of MIL-100(Al) and MIL-110(Al) are built up from discrete clusters in the form of trimers and octamers, respectively, which are linked through BTC ligands to form a 3D framework. Their cyclic stability significantly differs. The stability of MIL-100(Al) is mainly due to the stable *µ*_3_-O trinuclear units, where each aluminium atom in the trimer is bonded to four BTC functions and each trimer to six BTC ligands. Furthermore, at 140 °C, the structural water coordinating Al in the trinuclear unit is still present and therefore there are no coordinatively unsaturated Al sites [48]. Therefore, the desorption of water in the pores at this temperature has no effect on the core of the framework structure.

According to some literature [19], MIL-110(Al) is also classified into the group of hydrothermally stable MOFs (50% RH up to 300 °C). However, we found that under our experimental conditions (cycling treatment at 75% RH) it is unstable, since a complete loss of crystallinity and consequent formation of the AlO(OH) phase was determined. The aluminium octanuclear unit in MIL-110(Al) appears to be more sensitive to water attacks. Here, all eight Al in the unit are bonded to only nine carboxylate functions. Furthermore, the polarity of MIL-110(Al) SBUs, comprising the highest density of terminal OH and H_2_O ligands among the three tested structures, is very high. At such high humidity levels, a likely outcome of the penetration of water molecules to the octamers is breaking of the metal-ligand bonds and the hydrolysis reaction. Moreover, the large channels in MIL-110(Al) (16 Å) exceed the ones in MIL-100(Al) and in MIL-96(Al) structures. The hydrolysis of the framework may therefore occur at exposed metal-ligand sites in relatively low connected SBUs facing exceptionally large channels of MIL-110(Al), which provides no shielding from water attack.

## 4. Conclusions

In this work, three aluminium benzene-1,3,5-tricarboxylates MIL-96(Al), MIL-100(Al) and MIL-110(Al), which were synthesized from the same reagents and differed only in the dimensionality of the inorganic secondary building units and associated framework topologies, have been selected for the study of the effects of structural characteristics on their hydrothermal stability. We have shown that MIL-100(Al), which has already been recognized as a potential water adsorbent for heat transformation applications, has the highest water sorption capacity in comparison with the other two adsorbents and withstands 40 cycle-hydrothermal stability tests. Exceptional stability in humid conditions was also found for MIL-96(Al), whose loading after 40 cycles between 40 and 140 °C at 75% RH decreased only by 1% and whose structure did not collapse, although some degradation was observed. The detectable decrease of BET surface area and micropore volume is attributed to the formation of the boehmite AlO(OH) phase, which further limits the accessibility of the N_2_ molecules, as probe molecules in BET determination, in the interior of the pores. However, the hydrothermal stability of MIL-96(Al) is somehow expected due to the presence of corner sharing AlO_6_ octahedra in inorganic chains, which are additionally stabilized through a complex hydrogen bonding network. The results of DRIFT measurements revealed weak interactions between the linker and the water molecules, as well as reversible changes in the hydroxyls region during water adsorption-desorption cycles for all three studied frameworks. On the other hand, the obtained results revealed that MIL-110(Al) is hydrothermally unstable at selected humid conditions, as it did not retain its structure after hydrothermal stability tests. NMR and XRPD results coincide and they both confirm the formation of AlO(OH) during the cycling process. We concluded that the instability is due to the octanuclear clusters in the MIL-110(Al) framework being very susceptible to reaction with water, which further leads to structural decomposition.

Although further studies on the hydrothermal stability of MOFs are needed to fully understand all factors affecting their stability under humid conditions, the results of this study have provided additional insight into how the structure of the inorganic units and their accessibility affect their hydrothermal stability. It is proposed that secondary building units, well connected with the ligand molecules and/or additionally stabilized with intra-framework hydrogen bonds, increase the stability of Al MOFs.

## Figures and Tables

**Table 1:** Water loading data for all three studied materials after 20 and 40 cycles.

Material	Δwater Loading after 20 Cycles (%)	Δwater Loading after 40 Cycles (%)
MIL-96(Al)	−0.8	−0.8
MIL-100(Al)	−19	−20
MIL-110(Al)	−19	−25

**Table 2:** BET surface area, micropore and total pore volume of samples before and after water 40-cycling treatment obtained from N_2_ adsorption at 77 K.

Material	S_BET_^a^ (m^2^∙g^−1^)		V_micro_^b^ (cm^3^∙g^−1^)		V_total_^c^ (cm^3^∙g^−1^)	
	Before	After	Before	After	Before	After
MIL-96(Al)	310	50	0.12	0.02	0.13	/
MIL-100(Al)	1330	1770	0.57	0.63	0.64	0.85
MIL-110(Al)	780	/	0.32	/	0.34	/

^a^ BET surface areas calculated at p/p_0_ < 0.1, ^b^ micropore volumes obtained from Dubinin–Astakhov model (p/p_0_ > 0.01). and ^c^ total pore volumes calculated from N_2_ sorption isotherms at 77 K (p/p_0_ = 0.95).

## Data Availability

The supporting data are available on the journal webpage and from the authors.

## Supplementary Materials

The following supporting information can be downloaded at: https://www.mdpi.com/article/10.3390/nano12122092/s1; Figure S1: PXRD patterns of investigated materials; Figure S2: (a) Calculated powder XRD pattern of MIL-100(Al) without adsorbed water, (b) calculated powder XRD pattern of MIL-100(Al) with adsorbed water and (c) measured XRD pattern after hydrothermal experiment; Figure S3: SEM images of MIL-96(Al), MIL-100(Al) and MIL-110(Al) adsorbents before and after 40-cycles hydrothermal treatment; Figure S4: N_2_ adsorption isotherms of MIL-96(Al), MIL-100(Al) and MIL-110(Al) materials before 40-cycles hydrothermal treatment; Figure S5: N_2_ adsorption isotherms of MIL-96(Al), MIL-100(Al) and MIL-110(Al) materials in the relative pressure range below p/p^0^ < 0.2 before and after 40-cycles hydrothermal treatment; Figure S6: DRIFT spectra in the 5500–4500 cm^−1^ region of MIL-96(Al) heated at 200 °C (red) and cooled at 50 °C (blue) in a stream of humid air; Table S1: Isosteric heats of adsorption of MIL-96(Al), MIL-100(Al) and MIL-110(Al).
