# Supplementary material for: New Insight into Sorption Cycling Stability of Three Al-Based MOF Materials in Water Vapour
Source: Nanomaterials (Basel). 2022 Jun 17;12(12):2092. doi: 10.3390/nano12122092 (PMC9231181; doi:10.3390/nano12122092)
Supplement: Supplementary file 1 [file nanomaterials-12-02092-s001.zip › nanomaterials-1758268-supplementary.pdf]

nanomaterials

Supplementary Material

New Insight into Sorption Cycling Stability of three Al-Based MOF Materials in Water Vapour

Tadeja Birsa Čelič [1,†], Aljaž Škrjanc [1,2], Juan Manuel Coronado [3,‡], Tomaž Čendak [1,§], Victor Antonio de la Peña O'Shea [3], David Pedro Serrano [3,4] and Nataša Zabukovec Logar [1,2,*]

[1] National Institute of Chemistry, Hajdrihova 19, 1000 Ljubljana, Slovenia; tadeja.birsa@gmail.com (T.B.Č.); aljaz.skrjanc@ki.si (A.Š.); tomaz.cendak@gmail.com (T.Č.)
[2] School of Science, University of Nova Gorica, Vipavska 13, 5000 Nova Gorica, Slovenia
[3] IMDEA Energy Institute, Avenida Ramón de la Sagra, 3, Parque Tecnológico de Móstoles, 28935 Móstoles, Madrid Spain; jm.coronado@csic.es (J.M.C.); victor.delapenya@imdea.org (V.A.d.l.P.O.); david.serrano@imdea.org (D.P.S.)
[4] Chemical and Environmental Engineering Group, Rey Juan Carlos University, C. Tulipán, s/n, 28933 Móstoles, Madrid, Spain
* Correspondence: natasa.zabukovec@ki.si (N.Z.L.); Tel.: +386-1-476-03-71
† Current address: KRKA d.d., Šmarješka cesta 5, 8000 Novo Mesto, Slovenia.
‡ Current address: ICP-CSIC, C/ Marie Curie, 2. Cantoblanco, 28049 Madrid, Spain.
§ Current address: NLB d.d., Trg republike 1, 1250 Ljubljana, Slovenia.

1. X-Ray Powder Diffraction Analysis

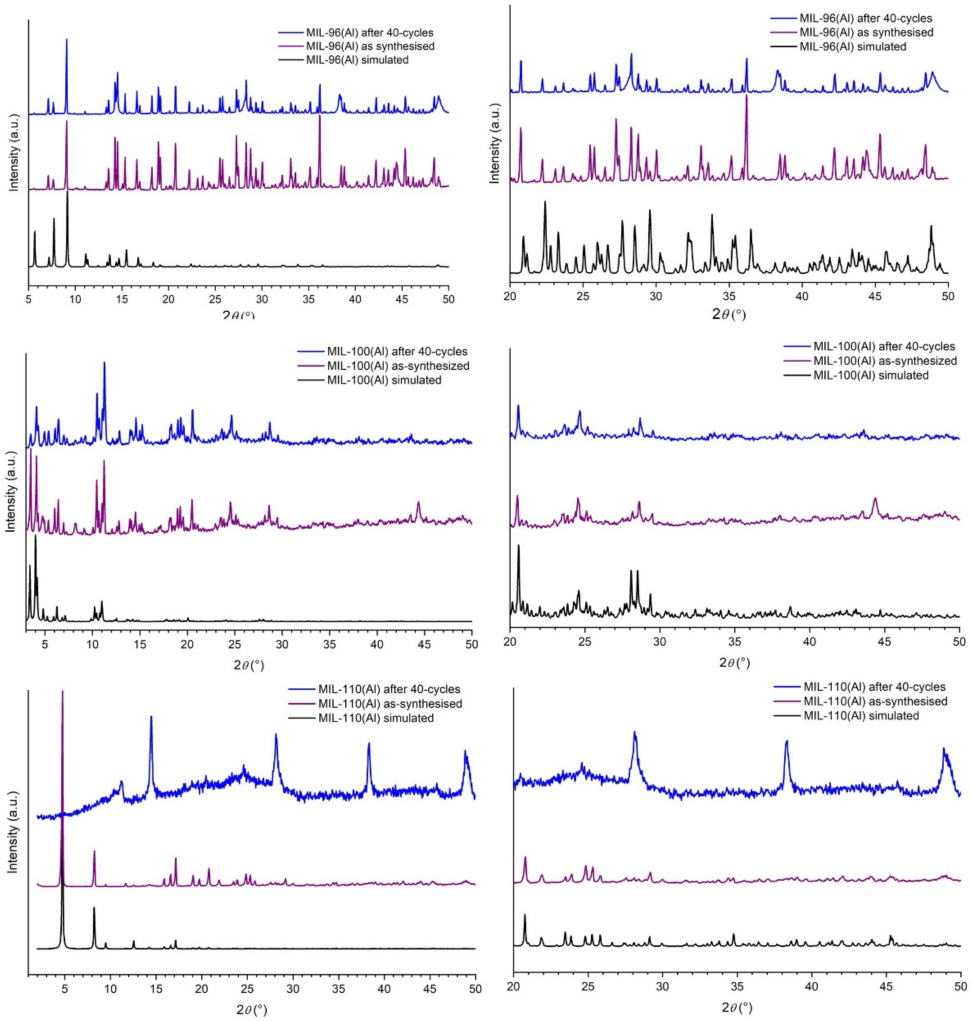

Figure S1. PXRD patterns of investigated materials in two 2θ ranges: simulated (black), as-synthesized (purple) and after 40-cycles hydrothermal treatment (blue). The intensity of the first peak in the XRPD patterns of MIL-96(Al) and MIL-100(Al) is dependent on the water content in the samples (see Figure S2).

As-synthesised samples of MIL-96(Al), MIL-100(Al) and MIL-110(Al), as well as MIL-100(Al) after 40-cycles test, are phase pure. Quantitative XRD analysis of MIL-96(Al) after 40-cycles hydrothermal treatment by using HighScore program 4.9 (Malvern Panalytical B.V., Almelo, The Netherlands) revealed the presence of 13 wt% of boehmite in the sample. MIL-100(Al) sample after 40-cycle treatment transforms into boehmite phase with traces of unknown phase (peak at ~11°2θ).

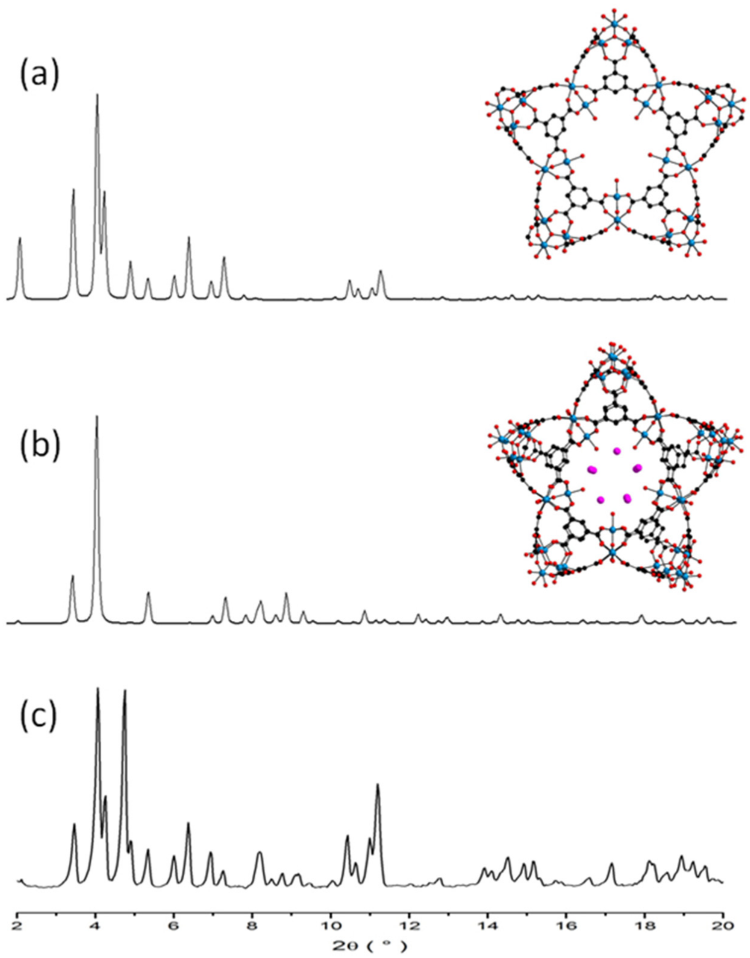

Figure S2. (**a**) Calculated powder XRD pattern of MIL-100(Al) without adsorbed water, (**b**) calculated powder XRD pattern of MIL-100(Al) with adsorbed water and (**c**) measured XRD pattern after hydrothermal experiment. The insets show MIL-100(Al) structure scheme viewed along the five-member ring opening with (**a**) empty pores and (**b**) with pores occupied with water molecules. Al atoms – blue dots, C atoms – black dots, O framework atoms – red dots, O adsorbed water atoms – purple dots.

Note that the positions of O-atoms belonging to adsorbed water in the structure scheme of Figure S2b are not refined and they do not represent the actual structure of the material after hydrothermal experiment as can be indicated by the comparison of the XRD patterns of Figure S2b and S2c. O atoms have been inserted manually just to show the impact on the diffraction peak intensities.

2. SEM Analysis

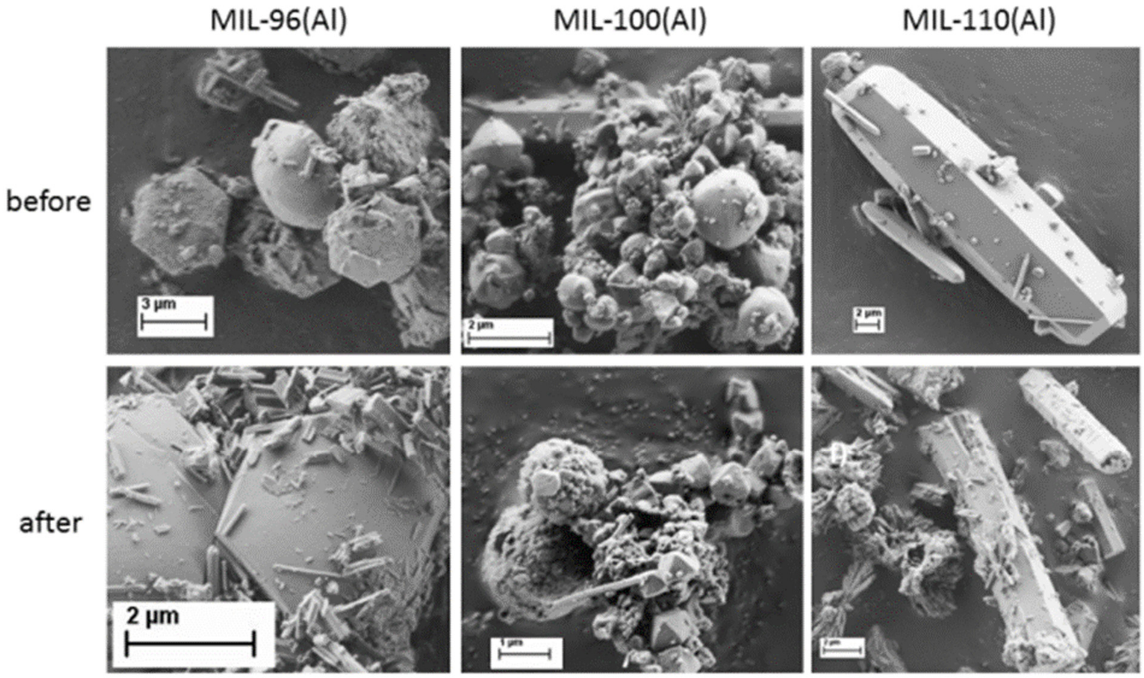

Figure S3. SEM images of MIL-96(Al), MIL-100(Al) and MIL-110(Al) adsorbents before and after 40-cycles hydrothermal treatment.

3. N_2-physisorption

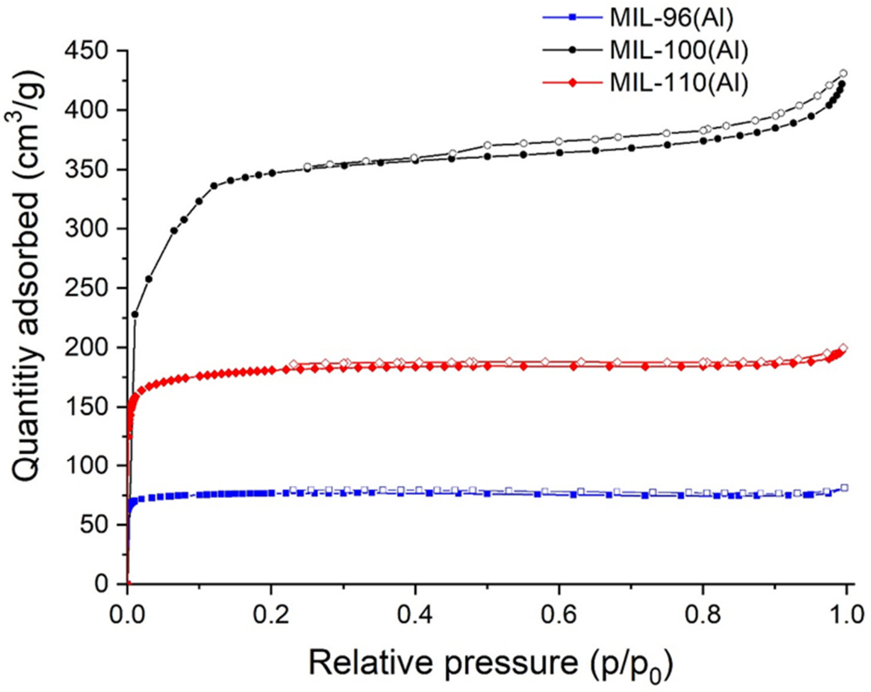

Figure S4. N_2 adsorption isotherms and pore size distribution of MIL-96(Al), MIL-100(Al) and MIL-110(Al) materials before 40-cycles hydrothermal treatment (top) and pore size distribution (bottom).

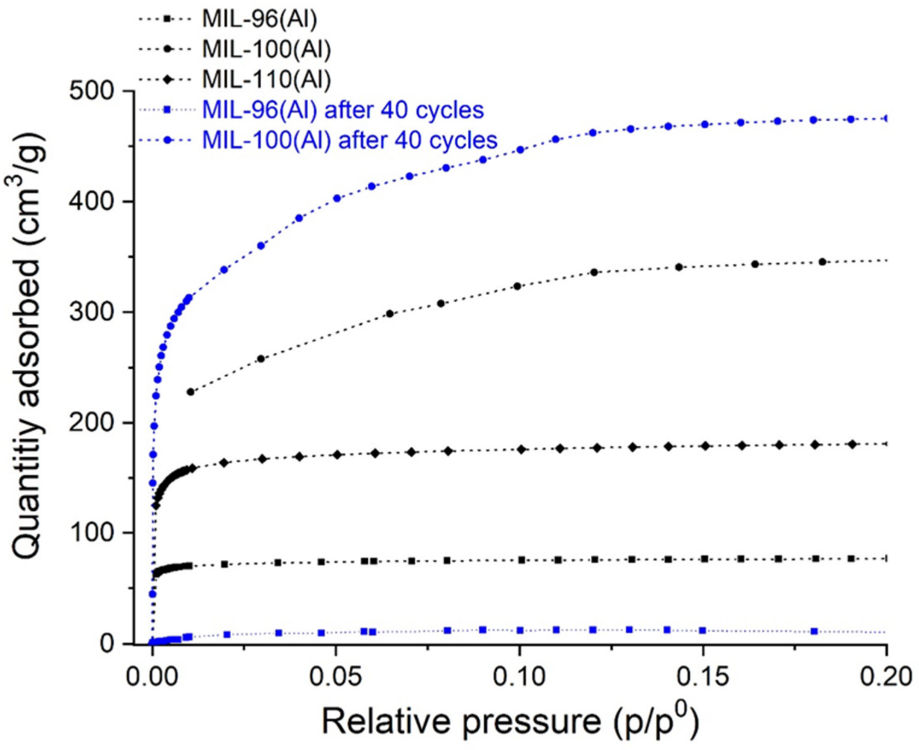

Figure S5. N_2 adsorption isotherms of MIL-96(Al), MIL-100(Al) and MIL-110(Al) materials in the relative pressure range below $p/p^0 < 0.2$ before and after 40-cycles hydrothermal treatment.

4. FTIR data

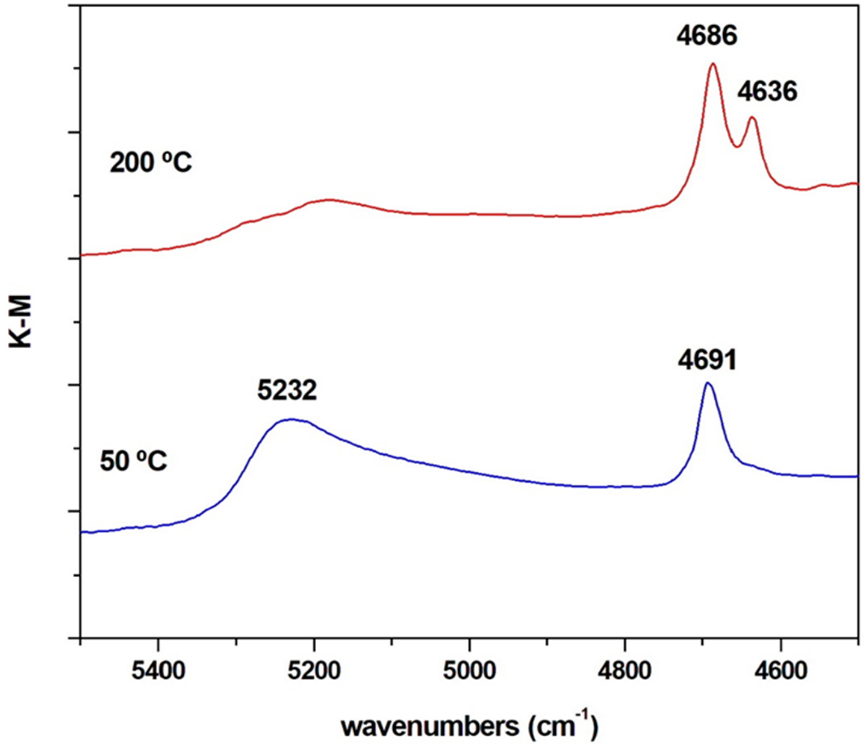

Figure S6. DRIFT spectra in the 5500–4500 cm^{-1} region of MIL-96(Al) heated at 200 °C (red) and cooled at 50 °C (blue) in a stream of humid air. The spectra show (ν+δ)combination bands of water (at around 5200 cm^{-1}) and hydroxyls groups (peaks at about 4600 cm^{-1}).

5. Isosteric heats of adsorption

Table S1. Isosteric heats of adsorption of MIL-96(Al), MIL-100(Al) and MIL-110(Al).

Material	p/p_0	βE_0 (kJ·mol^{-1})	q_{st} (kJ·mol^{-1})
MIL-96(Al)	0.1–0.2	10.8	54.2
	0.25–0.5	7.0	50.4
	0.55–0.7	6.4	49.8
MIL-100(Al)	0.1–0.2	8.5	51.8
	0.3–0.4	6.2	49.6
	0.5–0.7	5.4	48.7
MIL-110(Al)	0.1–0.2	9.9	53.3
	0.3–0.4	6.4	49.7
	0.5–0.7	6.7	50.0
